# A Comprehensive Ecotoxicological Evaluation of a Treated Olive Mill Wastewater and Obtained Sludge

**DOI:** 10.3390/toxics13080648

**Published:** 2025-07-30

**Authors:** José N. Pinto, Andreia Pereira, Ana Rita R. Silva, Diogo N. Cardoso, Amid Mostafaie, Fábio Campos, Iryna Rehan, Olga Moreira, Ivã Guidini Lopes, Daniel Murta, Alexandra Afonso, Margarida Oliveira, Karina S. Silvério, Maria Teresa Santos, Fátima Carvalho, Adelaide Almeida, Susana Loureiro

**Affiliations:** 1 Centre for Environmental and Marine Studies (CESAM) & Department of Biology, University of Aveiro, 3810-193 Aveiro, Portugal; jcpinto@ua.pt (J.N.P.); andreiafspereira@ua.pt (A.P.); dfilipe@ua.pt (D.N.C.); amid.mostafaie@ua.pt (A.M.); f.m.c@ua.pt (F.C.); sloureiro@ua.pt (S.L.); 2 National Institute of Agricultural and Veterinary Research (INIAV)—Polo de Inovação da Fonte Boa, Vale de Santarém, 2005-424 Santarém, Portugal; iryna.rehan@iniav.pt (I.R.); olga.moreira@iniav.pt (O.M.); 3 GeoBioTec Research Center, Faculdade de Ciências e Tecnologia, Universidade Nova de Lisboa, Campus da Caparica, 2829-516 Caparica, Portugal; 4 Centre for Interdisciplinary Research in Animal Health (CIISA), Faculty of Veterinary Medicine, University of Lisbon, 1300-477 Lisbon, Portugal; 5 Associate Laboratory for Animal and Veterinary Sciences (AL4AnimalS), 1300-477 Lisbon, Portugal; 6 Department of Biosystems and Technology, Swedish University of Agricultural Sciences, 23456 Alnarp, Sweden; iva.guidini.lopes@slu.se; 7 Ingredient Odyssey S.A.—EntoGreen, Rua Cidade de Santarém 140, 2005-079 Santarém, Portugal; daniel.murta@entogreen.com; 8 Egas Moniz Center for Interdisciplinary Research (CiiEM), Egas Moniz School of Health & Science, Caparica, 2829-511 Almada, Portugal; 9 Department of Applied Sciences and Technologies, Escola Superior Agrária de Beja, Instituto Politécnico de Beja, 7800-309 Beja, Portugal; alexandra.afonso@ipbeja.pt (A.A.); maguida111@hotmail.com (M.O.); kaarina_silveerio@hotmail.com (K.S.S.); t.santos@ipbeja.pt (M.T.S.); mfcarvalho@ipbeja.pt (F.C.); maalmeida@ipbeja.pt (A.A.); 10 Mediterranean Institute for Agriculture (MED), Environment and Development, University of Évora, 7006-554 Évora, Portugal; 11 Fiber Materials and Environmental Technologies (FibEnTech-UBI), Universidade da Beira Interior, R. Marquês de Ávila e Bolama, 6201-001 Covilhã, Portugal

**Keywords:** olive mill wastewater, chemical precipitation technique, wastewater treatment, ecotoxicological effects, soil safety, circularity

## Abstract

Olive mill wastewaters (OMWWs) are an environmental problem in the Mediterranean region, and it is crucial to explore strategies for their treatment and repurposing. The chemical precipitation technique (CPT) has been presented as a cost-effective wastewater treatment solution that might be applied to OMWW. The CPT-resulting precipitant subproducts (sludge) may be reprocessed (e.g., agricultural fertilizer and/or soil amendment), while the treated wastewater may be repurposed or reused (e.g., irrigation, aquaponic, or industrial processes). This study aimed to evaluate the efficacy of CPT in treating wastewater from the olive oil industry from an ecotoxicological perspective. Additionally, to assess the safe use of the obtained sludge in CPT treatment, its effects on soil biota were assessed. For this, a set of ecotoxicological assays using freshwater (*Raphidocelis subcapitata*, *Daphnia magna* and *Danio rerio*), terrestrial invertebrates (*Folsomia candida* and *Enchytraeus crypticus*), and plants (*Brassica oleracea* and *Lolium perenne*) were used as model organisms. Results demonstrated that CPT reduced OMWW toxicity to freshwater organisms, offering a favorable outlook on CPT’s potential as a wastewater treatment method. Increasing application rates of sludge in soil reduced the shoot biomass and the hydric content of both plants compared to the control. Survival of *F. candida* and *E. crypticus* was not affected by sludge in soil at any tested application rate, yet sludge application negatively affected the reproduction of both species, even at relevant sludge application rates (2%) of sludge in soils. Overall, the applicability of this sludge obtained by the CPT treatment in soils should be carefully evaluated due to the observed adverse effects on soil biota. Although the results of CPT were promising in reducing the toxicity of OMWW for these aquatic species, some adjustments/improvements should be performed to improve this technique and use all the obtained resources (treated water and sludge) in a fully circular perspective.

## 1. Introduction

In Mediterranean countries, olive oil production is a long-standing cultural tradition, but it also poses several environmental challenges [1]. Olive oil production entails the generation of vast amounts of solid waste in the form of an olive cake, commonly called olive pomace (OP), as well as high quantities of environmentally hazardous liquid waste in the form of olive mill wastewater (OMWW). OMWW physicochemical characteristics depend on a plethora of factors (e.g., climatic conditions of the olive tree, olive cultivars, degree of fruit maturation, storage time, and extraction process) [2]. Nevertheless, it is often possible to define OMWW as dark-colored, with an acidic pH [3], with high organic matter concentration [3], and the presence of recalcitrant and highly undegradable phytotoxic and antibacterial phenolic mixtures [4,5,6,7]. OMWW also has high concentrations of potassium (K), magnesium (Mg), and phosphate salts [3,8,9]. Nitrogen compounds, sugars, organic acids, and pectins are also highly present [3,10]. OMWW organic load is incredibly high [11], with correspondingly high levels of Chemical Oxygen Demand (COD) and Biochemical Oxygen Demand (BOD) [3,8].

In many Mediterranean countries, the discharge of OMWW is a prevalent problem [12]. Throughout the years, several treatment methods have been employed to treat OMWW, such as biological, chemical, and physical treatments; however, those methods might be inefficient and/or expensive [13,14,15,16,17,18]. The chemical precipitation technique (CPT) stands out as one of the possible methods for treating wastewater, which was presented as a potentially cheap, practical, and effective method to treat and reuse industrial wastewater [19]. Calcium hydroxide (Ca(OH)_2_) or slaked lime is added to influent water, resulting in immediate ion precipitation. Carbon dioxide (CO_2_) reacts with the supernatant, resulting in a spontaneous reaction. Metal ions are converted into hydroxide, sulfide, carbonates, or other less soluble compounds, while pH increases considerably and needs readjustment [20]. Prazeres et al. [21] demonstrated that calcium hydroxide precipitation decreased COD (11.4–17.8%), total phosphorus (23.6–45.9%), turbidity (60.9–100%), and phenols (26.9–48.0%) in OMWW. Despite this, water quality is variable; treated water is mostly free of coliforms and most suspended solids [22]. However, its use should be carefully evaluated considering the treated water’s available chemical characteristics, possibly being reused in many ways, such as irrigation, hydroponics, aquaponics, etc. [23]. In previous studies, the CPT was already used in treating an OMWW to reuse in the hydroponic production of lettuce [24]. Additionally, CPT produces dense sludges that may be reused and repurposed from a circular perspective. The sludge is a source of organic matter that may be used as a soil amendment to increase its pH and organic matter, add nutrients, increase aggregate stability, increase microbial biomass and water-holding capacity, and even decrease the bioavailability of metals [25,26]. In addition, it has already been proposed that sludge can be reused through bioconversion by some species of insects (e.g., *Hermetia illucens*) [27]. Therefore, the reuse and valorization of resources in a circular economic model can also be seen as a tool for waste management and become an important food and water security component, which can help to achieve the United Nations Sustainable Development Goal 6, “Clean Water and Sanitation” [17,28,29,30].

Nonetheless, the hazards related to the products from this CPT-treated wastewater and sludge must be fully assessed before large-scale application. Ecotoxicological assays, which provide essential insights into the bioavailable fraction of contaminants in the environment and complement chemical and biological analyses, have not yet been performed to fully evaluate CPT efficacy in OMWW. Therefore, this study focused on the ecotoxicological characterization of CPT-treated OMWW (CPT-OMWW) and the corresponding sludge produced on freshwater and terrestrial organisms, respectively. For that, (1) the CPT-OMWW was tested and compared with the untreated OMWW using a battery of ecotoxicological assays. The green microalgae *Raphidocelis subcapitata*, the crustacean *Daphnia magna*, and the fish *Danio rerio* were used as freshwater test model species and representative of different trophic levels; (2) the application of sludge in soils was tested by conducting ecotoxicological assays with four relevant organisms, namely, two soil model invertebrates, the springtail *Folsomia candida* and the oligochaete *Enchytraeus crypticus*, in addition to two different plant species, the dicotyledon agricultural species broccoli *Brassica oleracea* and the monocotyledon forage crop ryegrass *Lolium perenne*, exposed at various sludge application rates.

## 2. Materials and Methods

### 2.1. Chemical Precipitation Technique in Olive Oil Mill Wastewater (OMWW)

Olive mill wastewater was sampled from an olive oil industry located in Vale Vargo, Serpa, in the Alentejo region, Portugal. The OMWW was collected in plastic containers and transported to the laboratory for further characterization and treatment. The treatment applied to this water was the immediate one-step lime precipitation (herein named chemical precipitation technique—CPT), carried out according to Afonso et al. [24]. Briefly, a commercial hydrated lime—calcium hydroxide (Ca(OH)_2_, ≥95% purity) solution (200 g L^−1^) was used in the process, being added to a point at which the water’s pH reached 12.5 and a precipitate was formed. The treated water was left to rest for 48 h, and the supernatant was separated from the precipitate (sludge) by pumping, which was then stored in an open tank (high-density polyethylene (PEAD) container of 200 L), allowing natural carbonation by atmospheric CO_2_ for natural pH neutralization, 7.5 ± 0.2. The carbonation process took approximately 60 days. The reaction between the supernatant and CO_2_ induces calcium and magnesium elimination [21]. OMWW was stored at −20 °C, whereas CPT-OMWW was kept at 4 °C before testing. Before the ecotoxicity testing, the sludge was air-dried for five days before soil application. Both OMWW and CPT-OMWW were analyzed for pH, electrical conductivity (EC), Chemical Oxygen Demand (COD), Biochemical Oxygen Demand over 5 days (BOD_5_), total suspended solids (TSS), N Kjeldahl (Nt), phosphorus (Pt), dissolved oxygen (DO), ammonium (N-NH_4_), nitrate–nitrogen (N-NO_3_), nitrite–nitrogen (N-NO_2_), alkalinity and turbidity, as described in Afonso et al. and Ramalho et al. [24,31,32]. In addition, total phenols content was analyzed in both OMWW and CPT-OMWW water samples, according to the methodology described in Leouifoudi et al. [33]. The sludge was evaluated for total Ca, Na, Mg, Fe, Mn, and Zn by flame atomic absorption spectrometry (FAAS) according to NP EN ISO 6869:2007; P by UV-VIS according to NP 874:1998; N-Kjeldahl by digesting with sulfuric acid, followed by distillation and titration; ashes by NP ISO 5984:2014; dry matter by NP ISO 6496:2018; and microorganisms, namely *Escherichia coli* by ISO 16649-2:2001 and *Salmonella* spp. by ISO 6579:2002 [34,35,36,37,38,39].

### 2.2. Ecotoxicological Test Model Species

#### 2.2.1. Freshwater Species

*Raphidocelis subcapitata* inoculum was grown in the appLEE—applied Ecology and Ecotoxicology laboratory, Centre for Environmental and Marine Studies (CESAM), University of Aveiro (Portugal), in sterilized MBL—Medium Woods-Hole (autoclaved at 120 °C, at 1 bar, for 20 min) [40], under controlled conditions: continuous cool-white, fluorescent illumination (100 μE m^2^ s^−1^), at 20 °C ± 2. Prior to assembling the algae assays, 50 mL of *R. subcapitata* inoculum was prepared and grown under standard culture conditions for 3 days, being gently shaken once daily. After three days, the microalgae were harvested during the exponential growth phase, reaching a cell density of 1,456,286 cells/mL.

*Daphnia magna* K6 clones are cultured in the appLEE, CESAM, University of Aveiro (Portugal), in 800 mL of Standardized American Society for Testing and Materials (ASTM) hard water medium [41], at 20 °C ± 1, with a photoperiod cycle of 16 h light/8 h dark. Cultures are changed to clean media and fed 3 times a week, with a suspension of *R. subcapitata* 3 × 10^5^ algae cells/mL and a suspension of diluted and filtered seaweed extract (Marinure seaweed extract, provided by Glenside Organics Ltd., Hereford, UK). To check the sensitivity of culture daphnids, acute 24 h assays are conducted regularly, exposing daphnids to a reference toxicant (potassium dichromate (K_2_Cr_2_O_7_)). The 24 h lethal concentration inducing 50% mortality (LC_50_) values were always within the reference value for K_2_Cr_2_O_7_ (0.6 to 2.1 mg/L) [42].

*Danio rerio* eggs (wild type A.B.) were obtained from the Department of Biology Zebrafish facility at the University of Aveiro (Portugal). Zebrafish are kept in fish system water (FSW) in a ZebTEC (Tecniplast, Buguggiate, Italy) recirculating system, at 26 °C with a photoperiod of 14 h light/10 h dark. FSW comprises tap water purified by reverse osmosis, supplemented with salt (Instant Ocean Synthetic Sea Salt, Spectrum Brands, Blacksburg, VA, USA). Organisms are fed daily with dry flake food (Gemma Micro 500 (Skretting^®^, Burgos, Spain)). The holding tanks had a ratio of approximately 2:1 (male to female). Organisms are kept in a controlled environment: conductivity of 750 ± 50 μS/cm; pH 7.5 ± 0.5; DO of 95% saturation, salinity 0.35 ppt, temperature 26 °C ± 1.

#### 2.2.2. Soil Species

Both *Folsomia candida* and *Enchytraeus crypticus* are cultured in laboratory conditions of 20 ± 2 °C under darkness in the appLEE, CESAM, University of Aveiro (Portugal). *F. candida* are kept in plastic boxes with a mixture of plaster and activated charcoal (ratio 9:1) and fed twice a week with yeast [43]. *E. crypticus* are kept in plates with agar medium dissolved in ASTM and fed twice a week with a mixture of oat, dried yeast, yolk powder, milk, and fish oil. Cultures were kept under complete darkness at ≈20 ± 2 °C.

*Brassica oleracea* seeds were purchased from Rocalba, S. A. (Girona, Spain), and *L. perenne* from Flora Lusitana, Lda. (Cantanhede, Portugal).

### 2.3. Ecotoxicological Assays

#### 2.3.1. Freshwater and Soil Test Samples Preparation

Testing dilutions of both OMWW and CPT-OMWW were prepared by diluting testing samples with the respective organisms’ culture medium: MBL for *R. subcapitata*, ASTM for *D. magna*, and FSW for *D. rerio*. Concentrations of 0 (negative control), 6.75, 12.5, 25, 50, 75, and 100% of each test medium were used for all freshwater experiments. In the case of the OMWW, samples were defrosted at 4 °C prior to the experiments and acclimated to the respective temperature of the assay (both OMWW and CPT-OMWW).

Natural LUFA 2.2 soil (Speyer, Germany, www.lufa-speyer.de) was used in all soil-related ecotoxicological assays. LUFA 2.2 soil was incorporated (48 h before the experiments, as per OECD Guidelines for the Testing of Chemicals, no. 232 “Collembolan Reproduction Test in Soil”) [43] with different sludge application rates: 0 (negative control); 0.5; 1; 2; 4; 8% sludge (dry weight mass). At the start of each trial, the moisture content of each amended soil (as respective control) was adjusted to 50% and 60% Water Holding Capacity (WHC) for soil invertebrate and plant experiments, respectively. The intermediate application rate of 2% of sludge was selected considering the recommended application rate of sludge in soils used nowadays by the legal limits (40 ton/ha, Portuguese Decree Law Nº 276/2009), also evaluating lower (0.5 and 1%) and higher (4 and 8%) application rates for a complete evaluation [44]. Considering the legal limit of 40 ton/ha, the amount of sludge applied in test soils (in %) was calculated considering an incorporated depth of 15 cm and a soil bulk density of 1.33 g/cm^3^ for the reference soil (LUFA 2.2), according to Equation (1):

(1) *Soil amendment = Soil bulk density × Application rate × Depth*

where soil amendment is in g/cm^2^, soil bulk density in g/cm^3^, application rate in %, and soil depth in cm.

##### *Raphidocelis subcapitata* Bioassay

The growth inhibition assay was performed according to OECD Guidelines for the Testing of Chemicals, no. 201 “Freshwater Alga and Cyanobacteria, Growth Inhibition Test” [45], adapted by Eisentraeger et al. [46], using sterile 24-well microplates as test vessels. Absorbance (ABS) measurements were performed every 24, 48, and 72 h at 440 nm (Thermo Scientific Multiskan^®^ Spectrum, Waltham, MA, USA). Considering the dark color of the OMWW, ABS readings of blank samples (OMWW only) were performed. The aim was to ascertain if OMWW high turbidity affected the colorimetric method used in this bioassay. Eight wells at the center of the microplate were used for the experiment; the others were filled with Milli-Q^®^ ultra-pure water to prevent water loss from evaporation during the assay. Four replicates per dilution and eight for the negative control (MBL) were used. Each well was pipetted with 50 μL of algal inoculum at an initial concentration of 1 × 10^6^ cells mL^−1^ for an approximately initial inoculum of 50,000 algal cells. Plates were incubated for 72 h in an acclimatized chamber (KBWF 720 Binder, Darmstadt, Germany) under white, fluorescent illumination at 23 °C and were kept under continuous agitation (150–200 RPMs) for the test duration.

Blank plates were prepared to determine real algal absorbance by pipetting the tested dilutions of OMWW without any algal inoculum and placing them under the same conditions. Blank ABS was subtracted from the test’s ABS. Afterward, ABS values were converted into cell densities with Equation (2).

(2) Concentration cells mL−1=−17107.5+(ABS×7925350)

The average specific growth rate (Day^−1^) was determined with Equation (3) [45], with *Db* as the cell density at the end of the trial, *Da* as the cell density at the start, and *tb − ta* as the exposure time interval (72 h).

(3) $${Day}_{a-b\;}^{-1}=\frac{lnDb-\ln Da}{tb-ta}d^{-1}$$

##### *Daphnia magna* Bioassay

The *D. magna* bioassay followed the OECD Guidelines for the Testing of Chemicals, no. 202 “*Daphnia* sp. Acute Immobilization Test” [42]. To perform each acute toxicity bioassay, third to fifth brood neonates of *D. magna* k6 (age < 24 h) were collected from the cultures. The test was conducted inside 15 mL glass tubes, with 10 mL of test medium at a constant temperature of 20 °C ± 1 and a 16 h light/8 h dark photoperiod cycle. Four replicates per dilution with five organisms each were used. Additionally, a negative control of the ASTM solution was carried out. Daphnids’ immobilization was recorded after 24 and 48 h. EC, pH, and DO concentration were measured at the start and end of the experiment [42].

##### *Danio rerio* Bioassay

The *D. rerio* bioassay followed the OECD Guidelines for the Testing of Chemicals, no. 236 “Fish Embryo Acute Toxicity (FET) Test” [47]. Eggs, freshly fertilized by mature, properly acclimated fish, were thoroughly selected under a stereomicroscope (Stereoscopic Zoom Microscope-SMZ). All eggs that displayed abnormal development or malformations, coagulated, or showed unsuccessful fertilization were discarded. The test was performed in 24-well microplates with one egg per well. A volume of 2 mL of solution was used per well. Two dilutions of each sample were used for each microplate, divided in half, with ten wells each and four wells solely filled with FSW to confirm the test’s validity against microplate contamination. Thirty eggs were used per dilution (ten eggs/dilution/plate for three replicates total). Two additional 24-well microplates were used for negative (ASTM) and positive controls. Positive control followed the test design with four mg/L of 3,4-dichloroaniline concentration in FSW. The test had a total duration of 96 h. Eggs and embryos were checked daily for mortality, hatching rate, and developmental deformities (tail malformation, edema, delayed yolk sac absorption, scoliosis, lack of somite formation, and balance problems) as per the OECD 236 guideline [47]. EC, pH, and DO concentrations were measured at the start and the end of the experiment [47]. The temperature remained at 26 °C ± 1, with a 16 h light/8 h dark photoperiod cycle.

#### 2.3.2. Soil Bioassays

##### *Folsomia candida* Bioassay

*Folsomia candida* reproduction assay followed the OECD Guidelines for the Testing of Chemicals, no. 232 “Collembolan Reproduction Test in Soil” [43]. Ten age-synchronized juveniles (10–12 days) were placed in each test vessel with 30 g of soil/sludge mixture and controls. Jars were covered with perforated parafilm, randomly placed inside an acclimatized room (20 °C ± 1, 16:8 h light/dark photoperiod), and incubated for 28 days. During the test period, weekly, the moisture content was checked and replenished with water when necessary, and food was added (≈2 mg granulated dry yeast). At the end of the assay, each vessel was filled with water, transferred to a crystallizer dish, and then mixed to allow the organisms to float. Then the surface of the water was photographed, and the number of organisms (adults and juveniles) was counted using ImageJ software (version 1.43u) [48], evaluating adult survival and reproduction. pH and EC were measured at the start and the end of the test.

##### *Enchytraeus crypticus* Bioassay

The reproduction test with *E. crypticus* followed the OECD Guidelines for the Testing of Chemicals, no. 220 “*Enchytraeid* Reproduction Test” [49]. Ten adult organisms with well-developed clitellum were placed in each test vessel with 30 g of soil/sludge mixture and controls. The test had five replicates for each sludge application rate, including the control. The organisms were fed grounded oats, and the vessels were covered with perforated parafilm, then incubated in an acclimatized room (20 °C ± 1, 16:8 h light/dark photoperiod) for 21 days. Weekly, food was added, and water content was replenished when needed (based on weight loss). At the end of the test, vessels were filled with 96% ethanol and drops of a Bengal rose solution (1% solution in ethanol) to obtain the organisms’ coloration for proper visualization. The samples were stored in a 4 °C chamber for at least 24 h, and then the organisms (adults and juveniles) were counted. For this, the samples were sieved through a 125 µm mesh to separate the organisms from the soil. pH and EC were measured at the beginning and end of the test.

##### *Brassica oleracea* and *Lolium perenne* Plant Bioassays

The experimental procedure was similar for both plant species. The test procedure was adapted from the OECD Guidelines for the Testing of Chemicals, no. 208 “Terrestrial Plant Test: Seedling Emergence and Seedling Growth Test” [50]. Exposure was conducted inside an acclimatized chamber (KBWF 720 Binder, Tuttlingen, Germany) at 25 °C and 16:8 h light/dark photoperiod. Ten seeds were placed in the phytotoxic kits (MicroBioTests Inc., Mariakerke, Belgium) filled with soil/sludge mixtures (and control) to promote the visual monitoring of emergence, shoot, and root growth. For each sludge application rate, three replicates were used. pH and EC were assessed at the trial’s beginning and end.

Plants were allowed to grow for three days after at least 50% germination in control samples, and the germination index was calculated. This index is considered one of the most sensitive endpoints for assessing the phytotoxicity of soil amendments, and it is calculated according to Zucconi et al. [51] and Luo et al. [52] using Equations (4)–(6) subsequently.

(4) $$RSG=\frac{Number\;of\;germinated\;seeds\;(sample)}{Number\;of\;germinated\;seeds\;(control)}\times 100\%$$

(5) $$RRG=\frac{Total\;radicle\;length\;of\;germinated\;seeds\;(sample)}{Total\;radicle\;length\;of\;germinated\;seeds\;(control)}\times 100\%$$

(6) $$GI=\frac{RSG\times RRG}{100}$$

At the end of the trial (three days after seed germination), root and shoot sizes were measured and weighed (fresh weight). Both roots and shoots were dried in an oven (at 60 °C for 48 h); hydric content (HC) was assessed by using Equation (7).

(7) $$HC=\frac{FW-DW}{FW\;}\times 100$$

Fresh weight (FW), and dry weight (DW), expressed in milligrams (mg). HC is expressed in percentage (%).

#### 2.3.3. Statistical Analysis

Lethal concentration inducing a 50% mortality (LC_50_), and concentrations inducing a 50% effect (EC_50_) and respective standard errors (SE) were calculated by using nonlinear regression (three-parameter logistic curve) in Sigma Plot version 14.0 (Systat Software, Inc., San Jose, CA, USA, www.systatsoftware.com).

Normality and homoscedasticity were assessed using the Shapiro–Wilk’s test and Brown–Forsythe equal variance test in Sigma Plot version 14.0. Comparison between the different treatments and each test’s control was performed using a one-way ANOVA, with post hoc Dunnett’s test (*p* < 0.05). If data were not normally distributed, variances were not homogeneous, and data transformation did not correct either, a Kruskal–Wallis One Way Analysis of Variance on Ranks was performed, followed by post hoc Dunn’s method (*p* < 0.05).

## 3. Results and Discussion

### 3.1. Olive Mill Wastewater and Sludge Physicochemical Characterization

The physicochemical characterization of the OMWW and CPT-OMWW is presented in Table 1. As expected by the CPT treatment, the pH and EC increased with the addition of calcium hydroxide. These results are supported by previous studies using the same technique for similar samples of OMWW, even considering different OMWW samples [24]. Both the COD and BOD_5_ values decreased drastically—COD from 1504 to 238 mg L^−1^ and BOD_5_ from 35 to 2.7 mg L^−1^. This high decrease in COD shows the ability of CPT to remove organic matter. Regarding the total suspended solids (TSS), this value decreased almost seven times in the CPT-treated OMWW, showing an improvement in the water quality in terms of particles, which could have positive implications for different organisms, such as filter feeders (e.g., daphnids). As linked with TSS, turbidity decreased from 417 to 0.3 Nephelometric Turbidity Units (NTU). Considering the origin of this wastewater, olive oil industry wastewater, the presence of phenolic compounds should not be disregarded, as such, phenols were measured. The CPT reduced the concentration of phenols from 56 mg Gallic acid equivalents (GAE) L^−1^ to 13 mg GAE. L^−1^. This reduction was higher than the reductions in phenols in the study of Prazeres et al., where, after using the chemical precipitation technique, values were reduced between 25.9% and 48.0% in OMWW [21]. This reduction in phenols after CPT is mainly due to coagulants (in this case lime) that bind to phenolic compounds, causing them to form insoluble complexes that aggregate, forming the sludge that can be separated from the water after sedimentation [53]. Total nitrogen was almost completely removed after the CPT treatment, while phosphorus decreased 36 times from 84.3 to 2.33 mg L^−1^. After CPT treatment, OMWW accomplishes the Portuguese legal limits for irrigation reuse confirming its efficacy in terms of regulatory purposes, contrary to what was seen in untreated OMWW (Decree-Law No. 119/2019): BOD_5_ < 10 mg L^−1^; TSS < 35 mg L^−1^; total nitrogen < 15 mg L^−1^; Total phosphorous < 5 mg L^−1^; N-NH4 < 10 mg L^−1^) [54]. It is also relevant to mention that fecal coliforms, *E. coli*, and parasite eggs were completely removed from the wastewater by applying the CPT.

Although initially considered a residue, depending on its characteristics, the CPT-derived sludge might be applied in soil or can be used as an energy source or, for instance, of nutrients, such as nitrogen [55,56]. After CPT treatment, the obtained sludge was also characterized (Table 2). First, the sludge was dried at 65 °C before chemical analysis (data presented as dry matter). This sludge presented a basic pH (10), which could have advantages as a pH corrector in acid soils, e.g., mining soils. The high levels of Mg (4.09 g kg^−1^), Fe (8.87 g kg^−1^), and Ca (154.1 g kg^−1^) demonstrate the possibility of sludge being used in soils with a deficiency in these elements [57]. In addition, the CPT-sludge showed relatively high values of N-Kjeldahl—14.5 g kg^−1^, and low to no levels of microorganisms: *Salmonella* spp was absent, and *Escherichia coli* were < 1, which can be beneficial for agricultural purposes. However, it is noteworthy that due to the physical nature of the CPT sludge (very high moisture content, Ca, and Mg levels), it should probably not be considered fertilizer, but rather a soil amendment with specific application purposes.

### 3.2. Freshwater Ecotoxicity of Olive Mill Wastewater

#### 3.2.1. *Raphidocelis subcapitata*

The growth inhibition assays with *R. subcapitata* followed these OECD 201 validation criteria: average growth rates in the negative controls increased more than 16 times, and coefficients of variation did not exceed 7% at the end of the 72 h [45]. Both OMWW and CPT-OMWW displayed a decrease in the average growth rate with a decrease in wastewater dilution.

A ubiquitous characteristic of OMWWs is their high turbidity (Table 1) and dark coloring. The water matrix’s dark color affects light’s ability to permeate through the water, thus impairing any organism’s photosynthesizing ability [58]. Notwithstanding, after a series of dilutions with the organism medium (MBL), turbidity lessened, and negative responses could be considered and related to the presence of toxicants. After a preliminary test, the absorbance values of blank-only readings were obtained and calculated for the subsequent CV (using a value under the validation criteria threshold < 7%); 12.5% was the minimum concentration providing a reliable value not being significantly affected by the water’s high turbidity in OMWW and CPT-OMWW. Therefore, the results used in the present study are only related to the concentrations of 6.75% and 12.5% (Table 3). After 72 h at 12.5% OMWW, the average daily growth (day^−1^) of *R. subcapitata* decreased (Dunn’s, *p* < 0.05) by approximately 20% compared to the negative control. In CPT-OMWW, at the same concentration (12.5%), despite statistical significance (Dunn’s test, *p* < 0.05), the growth reduction was less evident than in the untreated OMWW, which shows that CPT reduces the impact on the algae growth rate.

Monomeric phenols are often cited as the most toxic components in OMWW [59], known for their phytotoxic [60] and antibacterial [5] characteristics. Based on this, the reduction in effects on algae growth in CPT-OMWW could be related to the decrease in the high phenol levels from untreated to treated OMWW (Table 1). Irrespective, OMWWs are deemed harmful to many species [2,61]. Even considering the limitations of using algae, as observed in the present study due to the colorimetric evaluation, *R. subcapitata* has already been used to evaluate OMWW toxicity [2]. At the same time, Andreozzi et al. showed that OMWW toxicity remained high for *R. subcapitata* after a combination of physicochemical processes (centrifugation–ozonation, centrifugation–solar photolysis, centrifugation–solar modified photoFenton, centrifugation–solar modified photoFenton–ozonation treatments) [62].

#### 3.2.2. *Daphnia magna*

**Figure 1:**
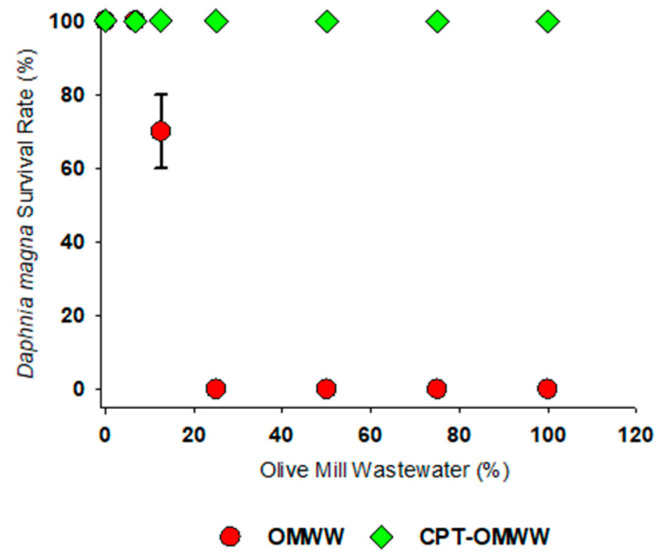
Survival (%) of *Daphnia magna* exposed to untreated olive mill wastewater (OMWW) and olive mill wastewater treated with the chemical precipitation technique (CPT-OMWW). The percentage (%) of values (mean ± standard error (SE)) is shown versus the tested water’s dilution.

In all the experiments with *D. magna*, mortality was less than 10% in the negative control (validity criteria). The chemical precipitation technique significantly reduced the acute toxicity (immobilization) in *D. magna* (Figure 1), as there was no evident toxicity in CPT-OMWW when compared to the untreated OMWW (LC_50_ of 12.7% (± 0.001 standard error (SE)).

The EC, pH, and DO values are presented in Table S1. The most concentrated samples (least diluted) of OMWW presented DO below 3 mg/L, established as the minimum OECD guideline 202. After treatment, CPT-OMWW DO was >3 mg/L in all dilutions, which can constitute one of the sources of toxicity induced by the OMWW, especially at low wastewater dilutions. For OMWW, low wastewater dilutions had a lower pH than the more diluted wastewater samples (pH varied from ≈8.05 at 0% of OMWW to ≈6.87 at 100% of OMWW), and EC increased at the most concentrated wastewater samples (least diluted) (≈558.33 µS/cm to ≈729.33 µS/cm). In CPT-OMWW, pH did not fluctuate throughout the concentrations (≈7.57 to ≈7.68), while EC significantly increased from 584.0 µS/cm in the ASTM control medium to 3290.0 µS/cm at 100% CPT-OMWW.

OMWWs are known to induce toxicity in *D. magna*. However, the toxicity levels of OMWW are highly dependent on their origin, either depending on the seasonal and geographical factors (type of soil, weather), extraction/processing methods, and/or phenolic content [61,63,64]. For instance, our calculated LC_50_ value was 12.7%, almost ten times higher than the 1.43% reported by Babić et al. for *D. magna* exposed to OMWW from Slovenian Istria, Slovenia [61]. Moreover, in the present study, following chemical precipitation treatment (CPT), acute toxicity was not observed in *D. magna*. This reduction in toxicity exceeds that reported by Isidori et al., who observed an 86% toxicity reduction in *D. magna* exposed to OMWW from Campania, Italy, treated with commercial microbial formulations [65]. As observed in Isidori et al.’s study, COD removal increased over time, which correlates with high dissolved oxygen in the medium and may explain the reduction in toxicity for *D. magna*, as observed in the present study [65]. Similarly, Oztekin & Sponza noted a significant decrease in OMWW toxicity to *D. magna* following wastewater sonication, attributing this to the degradation of phenols and phenolic metabolites in the wastewater [66]. As stated above, similarly, in the present study, the reduction in phenolic compounds with CPT treatment can be responsible for the reduction in untreated and CPT-treated OMWW. In addition to the presence of toxic compounds such as phenolic compounds in untreated OMWW, suspended solids may also induce adverse effects in daphnids mechanically, chemically, and biologically [67]. Tyagi et al. and Weltens et al. demonstrated that these solids can carry absorbed or bound compounds that induce both short-term and long-term toxicity in filter-feeding organisms like *D. magna* [68,69]. Apart from the mechanical toxicity induced by suspended solids in water to organisms, the interaction between those solids and chemicals dissolved in water can be a concern; for example, Herbrandson et al. observed synergistic toxic effects when suspended solids were combined with the pesticide carbofuran while evaluating the survival of *D. magna* [70]. High levels of total suspended solids were observed in the OMWW used in our study before treatment (Table 1). It is important to note that the aforementioned studies were conducted under short-term, acute exposure conditions. There is a critical need for further research involving long-term, chronic exposure to better understand the full spectrum of OMWW’s effects on *D. magna* and to supplement the findings of this study.

#### 3.2.3. *Danio rerio*

**Figure 2:**
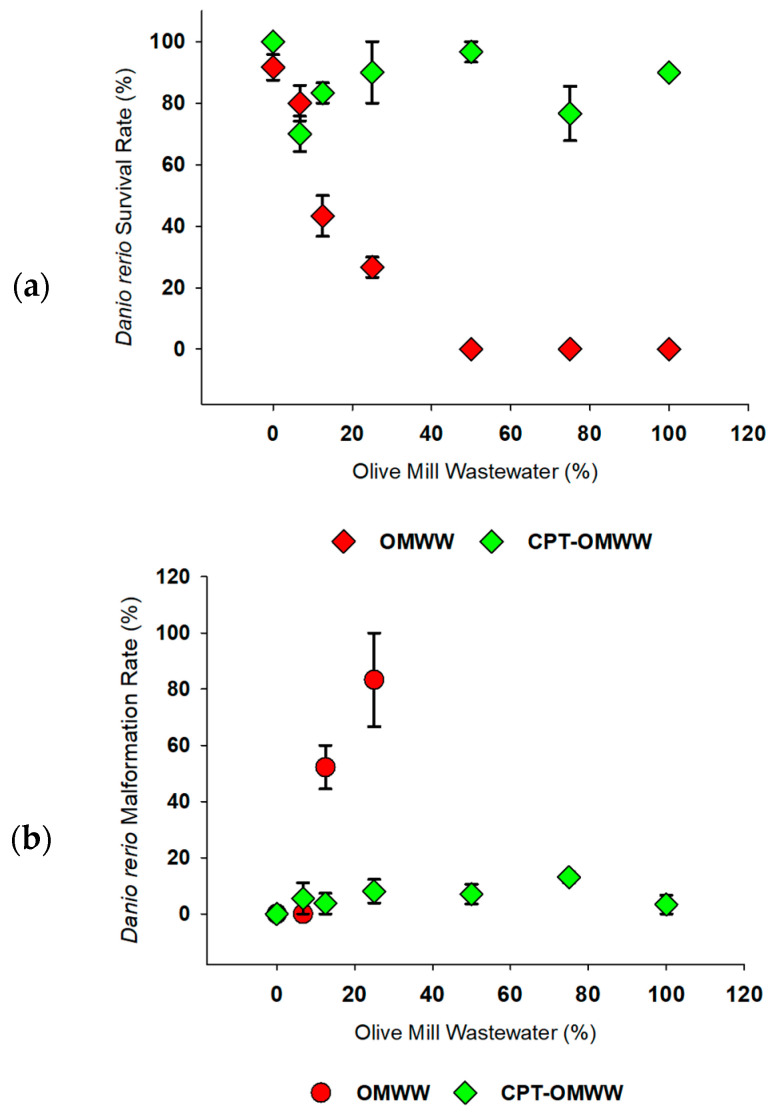
(**a**) Survival (%) after 96 h of *Danio rerio* exposed to untreated olive mill wastewater (OMWW) and olive mill wastewater treated with the chemical precipitation technique (CPT-OMWW). Percentage (%) (mean ± SE) of values is shown versus olive mill wastewater concentration (%). Malformations in the three highest concentrations of OMWW are not presented in the figure due to 100% mortality. (**b**) Malformations (%) after 96 h of *D. rerio* exposed to untreated OMWW and CPT-treated OMWW. Percentage (%) (mean ± SE) of values is shown versus olive mill wastewater dilution (%).

Both negative and positive controls followed the OECD 236 [47] validation criteria: the embryos’ survival in the negative control was higher than 90%, while in the positive control, the survival was below 70%. For the statistical analysis, only the negative control was taken into consideration. After CPT treatment of the OMWW, *D. rerio* mortality was reduced when compared to OMWW, where *D. rerio* in CPT-OMWW presented mortality close to zero, and in OMWW presented an LC_50_ value of 13.89% (±1.01 SE) (Figure 2a). Hatching rates closely followed survival rates for both tested wastewaters (OMWW = EC_50_ of 13.7% (± 0.96 SE)) and no evident toxicity after CPT. OMWW induced a high rate of malformations at 12.5 and 25% of the wastewater, with a mean value of 83.33% (± 11.78 SE) at 25% of OMWW (Figure 2b). Only 96 h post-fertilization (hpf) living organisms were considered for malformation data analysis, excluding the results for the 50–100% of wastewater due to total mortality of the organisms.

The conductivity, pH, and DO concentration results are presented in Table S2. The three physicochemical parameters followed closely with the values obtained during the *D. magna* assay. DO levels were low in OMWW; at the highest concentrations of OMWW, DO was < 3 mg/L (Table S2), which is below the advised in the OECD guideline 236 [47]. EC decreased at higher concentrations in OMWW; however, CPT-OMWW EC values were high (100% CPT-OMWW = 3331.9 µS/cm). EC levels are above *D. rerio*’s optimal range [71], so they could account for malformations during embryonic development. Before CPT, pH decreased with increasing concentrations (≈7.77 to ≈6.91), while following CPT, the effect on the pH was reversed (≈8.29 to ≈8.35).

The results observed for *D. rerio* mirrored those obtained for *D. magna* and might be considered positive for CPT as a wastewater treatment method. CPT treatment in OMWW incurred a decrease in the mortality of both organisms. Like *D. magna*, the levels of toxicity in *D. rerio* are dependent on the origins of the OMWW. Both Rouvalis et al. and Babić et al. exposed *D. rerio* embryos to untreated OMWW and obtained different LC_50_ values (0.48% for the former and 7.05% for the latter), both of which are lower than those obtained in the present study. Regarding the development of malformations, CPT greatly reduced the rate of malformation development in surviving embryos [55,72]. Wu et al. consider metals (e.g., zinc, copper, cadmium, and manganese) present in OMWW as the key components for malformation development in *D. rerio* embryos due to their ability to permeate the chorion [73]. Considering that CPT treatment is often cited as capable of reducing metal content in wastewater [74], it can explain the reduction in effect, although the metals were not evaluated in the present study. However, it cannot be rejected that these metals might have synergistic effects with other compounds, especially phenolic compounds, thus increasing the OMWW toxic potential [61].

### 3.3. Soil Ecotoxicity After Sludge Application

#### 3.3.1. *Folsomia candida* and *Enchytraeus crypticus*

For the soil invertebrate assays, both controls followed the OECD’s validation criteria [43,49]. Adult mortality was <20% for both species in the negative control—no sludge application. Juveniles per replicate were >100 (mean value of 777) and >50 (mean value of 691) for *F. candida* and *E. crypticus*, respectively, and the number of juveniles’ coefficient of variation was <30 (5.6%) and <50% (6.1%), respectively.

**Figure 3:**
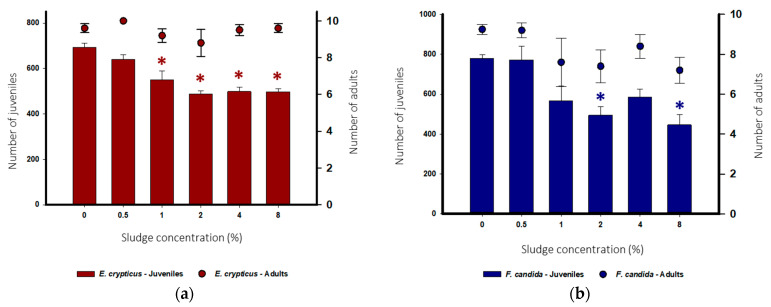
(**a**) *Enchytraeus crypticus* reproduction (Left y-axis) and adult survival (Right y-axis) after 21 days, and (**b**) *Folsomia candida* reproduction (Left y-axis) and adult survival (Right y-axis) after 28 days exposed to different sludge application rates using different concentrations in soil (%). All values are presented as mean ± SE, and asterisks (*) signify statistical differences in comparison with the control (0%) (Dunnett’s, *p* < 0.05).

Sludge application in soil did not negatively affect adult *E. crypticus* survival (Figure 3a). However, *E. crypticus* reproduction was significantly reduced at 1%, 2%, 4%, and 8% of sludge application (Dunnett’s, *p* < 0.05, Figure 3a). Likewise, sludge application did not negatively affect adult *F. candida*’s survival at any of the tested applications. Notwithstanding, *F. candida* reproduction was significantly affected by the increase in sludge in soil, with a statistical reduction in the number of juveniles produced at 2% and 8% sludge in soils compared with the control (Dunnett’s, *p* < 0.05, Figure 3b). The EC and pH results are presented in Tables S3 and S4. For *E. crypticus*, higher sludge application rates increased pH (≈5.76 to ≈7.56). A similar effect was seen in the EC (≈190.0 µS/cm to ≈278.67 µS/cm). *F. candida* results mirrored those obtained for *E. crypticus* (pH ≈ 5.5 to ≈7.6 and EC ≈ 179.3 µS/cm to ≈223.0 µS/cm).

Both organisms were similarly affected by the presence of sludge in soils, with no statistical differences in adult survival and decreased reproduction rates. Considering that the studied sludge is a precipitate obtained from the CPT in OMWW, even if all the contaminants are not addressed, it is assumed that, eventually, toxic compounds in wastewater are deposited in the sludge. This is why this sludge should be ecotoxicologically evaluated since it is known that contaminants or a mixture of contaminants in OMWW-originated sludge (e.g., evaporation ponds) are present and could adversely impact terrestrial organisms [75,76]. To our knowledge, there are no published studies on sludge toxicity obtained using CPT on OMWW on soil invertebrates. The evaluation of sludges obtained from wastewaters originating from an olive industry in Mira, Portugal, although using a different treatment (biological treatment), was already assessed by Natal-Da-Luz et al. [77]. Applying different application rates (0%, 0.4%, 1%, 1.6%, and 3% of sludge), the authors concluded that the sludge did not negatively affect *F. candida* reproduction at the tested application rates [77]. In fact, at the higher application rates (1%, 1.6%, and 3%), *F. candida* reproduction significantly increased compared to the control soil (that used field-collected soil from the suburban limits of the city of Coimbra, Portugal). According to the authors, this might be related to increased organic compounds in the sludge, which could promote favorable conditions for the reproduction of these species. A significant increase in the reproduction of *Eisenia fetida* was observed at the application rate of 1% relative to the control. However, there are no significant effects at higher sludge application rates (1.6% and 3%) compared to the control [77]. Those results contrast with the ones obtained in the present study, where further toxic effects were found, even considering the higher application rates tested here. It is challenging to compare the results from different studies since, for example, the source of the originating sludge is different, namely the type of wastewater and treatment technique. This would make a difference in terms of which (toxic) compounds can be allocated to sludge at the end of the treatment process. Phenolic compounds might be a plausible cause of the decrease in both species’ reproduction, as seen in previous studies with collembolans [78]. Prazeres et al. demonstrated that calcium hydroxide precipitation can reduce the total phenols present in a water sample; however, those phenolic compounds will then become a part of the precipitated sludge [21]. The phenolic compounds were not assessed in the obtained sludge in the present study. Nevertheless, high levels of phenols were confirmed in the OMWW, which were significantly reduced after CPT treatment (Table 1); this indicates their transference to the sludge by deposition. The degradation of phenolic compounds is not a simple process, and these compounds remain in the environment for prolonged periods, with a few chemical and biological treatments being effective for their removal [79]. However, to the authors’ knowledge, no studies were conducted to verify the potential of the CPT to degrade phenolic compounds, which should be investigated further. Another important factor to consider is the reference soils used for amending the different sludges. As previously stated, the reference soil used in Natal-da-Luz et al. was an agricultural soil known to be free of fertilizers and pesticide application for over 5 years [77]. Our study used LUFA 2.2, a natural soil with different characteristics, such as lower pH and low cation exchange capacity. These varying physicochemical characteristics can affect the bioavailability of different compounds (e.g., metals), potentially increasing toxicity to soil organisms [80].

#### 3.3.2. *Brassica oleracea* and *Lolium perenne*

Germination indexes (GI) were expressed in percentages and shown in Figure S1. Although a slight increase in GI was observed for *B. oleracea* compared to the control, sludge application in soil showed no statistically significant differences at all tested application rates. For *L. perenne*, no significant differences were observed for the GI.

**Figure 4:**
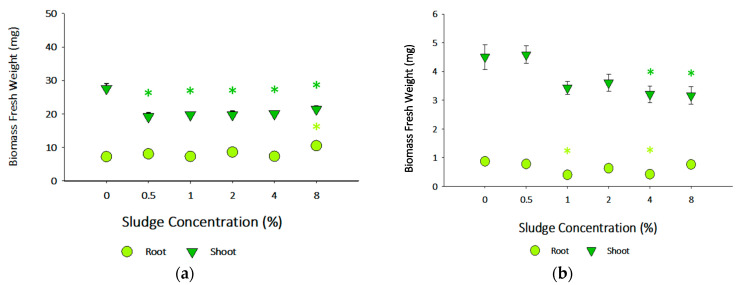
Roots and shoot biomass (mg, fresh weight, FW) of (**a**) *Brassica oleracea* and (**b**) *Lolium perenne* after exposure to different application rates using different concentrations of sludge in soil (%). Biomass value (mg) (mean ± SE) is shown versus sludge concentration (%). Asterisks (*) indicate statistical differences to the respective control (0%) (Dunnett’s, *p* < 0.05) for both root and shoot biomass of *B. oleracea* and shoot biomass of *L. perenne* (Dunn’s, *p* < 0.05) for root biomass of *L. perenne.*

The effects of sludge on the shoots and roots biomass of *B. oleracea* and *L. perenne* (Fresh weight, FW) are shown in Figure 4. Sludge application significantly reduced the shoot biomass of *B. oleracea* exposed to all sludge-tested application rates compared to the control soil. At the same time, the root biomass of *B. oleracea* increased only at the 8% sludge application when compared to the control soil (*Dunnett’s*, *p* < 0.05). Plants may prioritize root growth over shoot growth to, for instance, increase nutrient uptake under stress conditions, as observed for *B. oleracea* in the present study, in line with the observations by Arif et al. for other species of *Brassica* under salinity stress, where high levels of salts were also present [81]. *L. perenne’s* shoot biomass decreased at 4% and 8% of sludge application (Dunnett’s, *p* < 0.05), whereas root biomass decreased at 1% and 4% of applied sludge when compared to the control soil (Dunn’s, *p* < 0.05). The root biomass and length responses of *L. perenne* were different, but it should be noted that one species is dicotyledonous (*B. oleracea*), and the other is monocotyledonous (*L. perenne*), with different architecture in terms of root systems and thus plasticity [82].

**Figure 5:**
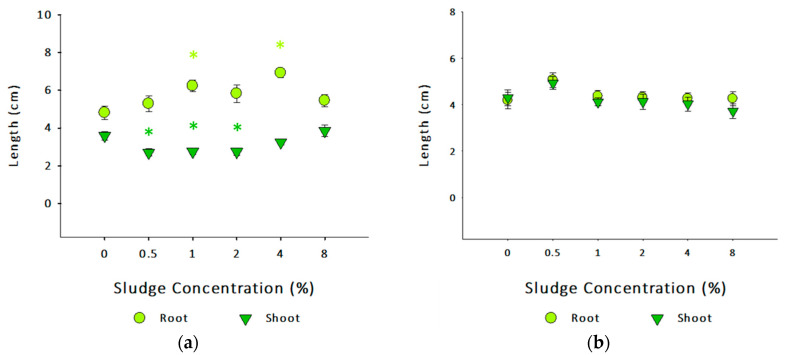
Root and shoot length (cm) of (**a**) *Brassica oleracea* and (**b**) *Lolium perenne* after exposure to different concentrations of sludge. Length value (cm) (mean ± SE) is shown versus sludge concentration (%). Asterisks (*) denote statistical differences from the respective control (0%) (Dunnett’s, *p* < 0.05).

Shoot and root length for both species did not follow a well-defined concentration-dependent response after sludge application (Figure 5). For *B. oleracea*, shoot length decreased at 0.5, 1, and 2% of sludge application compared to the control (Dunnett’s, *p* < 0.05). At the same time, sludge application significantly increased root length at 1% and 4% compared to the control (Dunnett’s, *p* < 0.05). For *L. perenne*, neither shoot nor root length was significantly affected after sludge application. These different responses observed in this study could be challenging to integrate. It is well-reported that many factors can influence plant growth. These include nutrient bioavailability, soil electrical conductivity, microbial communities, the presence or absence of particular biota, and hazardous constituents [83,84,85]. Although the present study did not include soil microbial community analysis, such data would be pertinent to understand the impact of sludge application on soil health and functioning, which will have an impact on plant health. Nutrient availability decreases with soil depth, so plants naturally grow shorter root systems. On the other hand, water availability often increases with depth, necessitating an extended root system from the plants [86]. Understandably, plants under stressful/growth-limiting conditions would minimize the energetic costs needed to develop more extensive and longer fragments [87].

**Figure 6:**
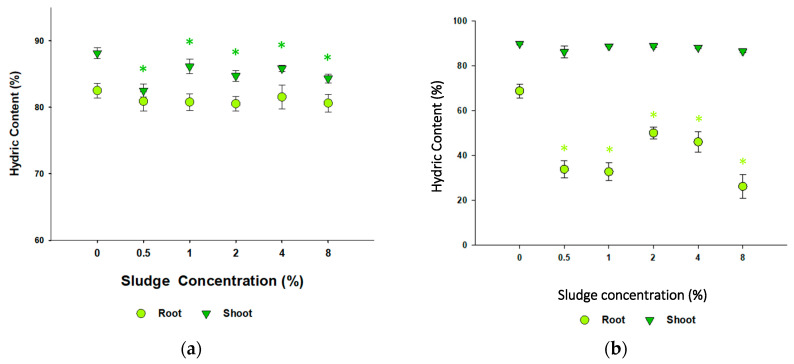
Root and shoot hydric content (%) of (**a**) *Brassica oleracea* and (**b**) *Lolium perenne* after exposure to different concentrations of sludge. Hydric content (%) (mean ± SE) is shown versus sludge concentration (%). Asterisks (*) denote statistical differences to the respective control (0%) (Dunnett’s, *p* < 0.05).

Sludge application significantly decreased the shoot hydric content in *B. oleracea* at all tested application rates (Dunn’s, *p* < 0.05) (Figure 6). However, for *L. perenne*, statistically significant differences were observed only at 4% and 8% sludge application (Dunnett’s, *p* < 0.05). The results of EC, pH, and DO concentration are presented in Tables S5 and S6. For *B. oleracea*, higher sludge application concentrations increased pH (≈5.7 to ≈7.5). A similar effect was seen in the EC (≈185.9 µS/cm to ≈255.0 µS/cm). *L. perenne* results mirrored those obtained for *B. oleracea* (pH ≈ 6.4 to ≈8.1 and EC ≈ 191.7 µS/cm to ≈226.3 µS/cm).

Similarly to phytotoxicity, a critical aspect of olive mill wastewater, it is essential to determine the effects of applying sludge to soil on different plant species. To the authors’ knowledge, there is a lack of information regarding phytotoxicity studies with soil–sludge amendments obtained from the olive mill industry; however, there is information regarding the direct application of low concentrations of liquid, untreated OMWW to soil. Although both matrices (liquid OMWW vs. sludge) are different, and the bioavailability for plants may be different as well, this comparison is the only one possible, considering the very limited information available in the literature. Rusan & Malkawi exposed the monocotyledon *Zea mays* to soils containing different concentrations of a liquid OMWW and demonstrated that low OMWW concentrations induced a positive response in the plant species’ growth (at 25% concentration), while higher concentrations (50%, 75%, and 100%) decreased growth [88]. These results might be related to higher phytotoxic phenolic compounds at high concentrations, resulting in a negative response with a higher OMWW application. At the same time, at lower concentrations (up to 25% concentration), phenolics were not phytotoxic, and the OMWW exposure increased the availability of various essential nutrients [89]. Moreover, Lanza et al.’s study demonstrated that long-term (9 years) controlled spreading of liquid OMWW had positive effects on the growth of an olive orchard, increasing the uptake of nitrogen (N), phosphorus (P), potassium (K), and organic matter, as well as a benefit on the soil properties [90]. Our results reflect the findings of Lanza et al., where an increase (although not significant) in the germination index was found after sludge application in soils, when compared to the control soil [90]. Contrariwise, the significant decrease in the root and shoot biomass, as well as the fluctuation in hydric content of the two species (at higher sludge application rates), are more in line with the findings of Rusan & Malkawi under higher liquid OMWW concentrations [88].

For the present work, the presence of hazardous contaminants in sludge (e.g., monomeric phenols) should not be disregarded, considering the source of the obtained sludge (OMWW treatment) and the reported high levels of phenols that could inhibit plant growth. Chatterjee & Chatterjee also showed that high metal concentrations affect *Brassica oleracea*’s transpiration rate and the stomate opening in plants, directly reflecting this work’s findings, as a higher transpiration rate will result in an increased water loss from the plant, thus leading to a lower hydric content [91,92].

The use of different wastewaters (used either as raw material, weathered, or treated with different methods) and/or the respective produced sludges has been used in soils as organic amendments or in agricultural lands to improve soil productivity [93,94,95,96]. Specifically for OMWW, it was shown to be a source of nutrients (such as N, P, K, Mg, and Fe) and organic matter, with potential benefits for soils (e.g., increase in soil organic matter, nutrient availability, and improvement in physical properties) and crops (e.g., an increase in nutrients, plant growth/biomass, and anti-microbial and anti-fungal properties) [93]. As shown by Iticescu et al., sludges contain high nitrogen, phosphorus, and organic matter content, providing good properties as organic fertilizers [95]. Increasing the knowledge and use of alternative organic fertilizers is crucial for increasing productivity sustainably; however, their safety should be carefully evaluated. For instance, in the present work, in the 2% application rate of sludge (the legally recommended amount according to Decree-Law Nº 276/2009), both the plant and soil invertebrate test results revealed that the sludge usage as a generalized soil amendment or fertilizer may not be recommended [44]. However, other potential uses may be possible for this sludge type, such as being more focused on soil amendment for highly acidic soils with no agricultural purpose, to be converted by insects, and as a source of energy and other resource recovery, such as carbon and metal recovery (e.g., for construction materials), which is of great importance in the context of circular economy procedures [55].

## 4. Conclusions

Overall, considering the tested species, the chemical precipitation technique was shown to decrease the toxicity of the olive mill wastewater used in the present study. The most significant ecotoxicity reduction was observed for *D. magna* and *D. rerio*, with no to minimal toxicity after CPT treatment of OMWW. Considering this complex matrix, the initial OMWW source of toxicity is difficult to perceive. Besides the complexity of chemicals present, dissolved oxygen, conductivity, and pH are among the properties that contribute to OMWW toxicity. At the same time, the produced sludge did not affect *F. candida* and *E. crypticus* adult survival. However, there was a reduction in the reproduction of both soil invertebrate species at higher application rates and a decrease in plant shoot biomass and length; hence, the results should be carefully interpreted. The approach used in the present study shows that decisions on the use of OMWW, treated OMWW, and respective sludge in aquaculture, agriculture, or other practices must be carefully made while integrating physicochemical, biological, and ecotoxicological assays due to the complexity of the matrices. Therefore, legislation should be improved so that the demand regarding these complex matrices goes beyond chemical and microbiological analysis.

## Figures and Tables

**Table 1:** Physico-chemical characterization of the raw olive mill wastewater (OMWW) collected from an olive oil processing plant and after the chemical precipitation technique (CPT-OMWW), values are presented as mean with standard deviation (*n* ≥ 3).

Physico-Chemical	OMWW Sample	CPT-OMWW Sample
pH	6.5 ± 0.10	7.8 ± 0.02
EC (mS cm^−1^)	0.7 ± 0.003	2.6 ± 0.004
COD (mg L^−1^)	1504 ± 350	238 ± 45
BOD_5_ (mg L^−1^)	35 ± 3	2.7 ± 0.6
TSS (mg L^−1^)	222 ± 0.6	32 ± 0.2
N_t_ (mg L^−1^)	439 ± 7	0
P_t_ (mg L^−1^)	84.3 ± 4.2	2.33 ± 010
DO (%)	5.6 ± 0.8	20.5 ± 0.5
Alkalinity (CaCO_3_ mg L^−1^)	0	17,676 ± 69
Turbidity (NTU)	417 ± 5	0.3 ± 0.03
N-NH_4_ (mg L^−1^)	234 ± 1	0
N-NO_3_ (mg L^−1^)	2.5 ± 0.1	0.8 ± 0.1
N-NO_2_ (mg L^−1^)	0.018 ± 0.00	0.003 ± 0.00
Phenols (mg GAE L^−1^)	56 ± 6	13 ±2
Fecal Coliforms (Nº/100 mL)	93 ± 2	0
*E. coli* (Nº/100 mL)	9.1 ± 0.2	0
Parasite eggs (Nº/L)	9.1 ± 0.1	0

EC—Electrical Conductivity; COD—Chemical Oxygen Demand; BOD—Biochemical Oxygen Demand over 5 days; TSS—Total Suspended Solids; DO—Dissolved Oxygen; GAE—Gallic acid equivalents; NTU—Nephelometric Turbidity Units.

**Table 2:** Chemical and biological characterization of the sludge obtained after the chemical precipitation technique (CTP-sludge) in the olive mill wastewater.

Chemical and Biological Characteristics	CPT-Sludge Sample
pH	10
Ca (g kg^−1^)	154.1
P (g kg^−1^)	1.22
Na (g kg^−1^)	0.962
K (g kg^−1^)	1.44
Mg (g kg^−1^)	4.09
Cu (g kg^−1^)	0.117
Fe (g kg^−1^)	8.87
Mn (g kg^−1^)	0.099
Zn (g kg^−1^)	0.54
N-Kjeldahl (g/kg^−1^)	14.5
Ashes (%)	47
Dry matter (%)	94.1
*Escherichia coli* (number/g)	<1
*Salmonella* spp./50 g	Not present

**Table 3:** Average daily growth (Day^−1^) after 72 h of *Raphidocelis subcapitata* exposed to 0, 6.75, and 12.5% of OMWW and CPT-OMWW, and growth reduction (%) compared to the negative control (MBL solution). “-” not determined. Asterisk (*) represents significant differences when compared to control (0) (Dunn’s test, *p* < 0.05).

	OMWW		CPT-OMWW	
Wastewater (%)	Average Daily Growth (Day^−1^)	Growth Reduction (%)	Average Daily Growth (Day^−1^)	Growth Reduction (%)
0	1.462 ± 0.006 SE	-	1.371 ± 0.010 SE	-
6.75	1.322 ± 0.025 SE	9.55	1.349 ± 0.025 SE	1.57
12.5	1.174 ± 0.089 SE *	19.67	1.337 ± 0.089 SE *	2.43

## Data Availability

Data is available at Zenodo: https://doi.org/10.5281/zenodo.14591706 (accessed on 28 July 2025).

## Supplementary Materials

The following supporting information can be downloaded at: https://www.mdpi.com/article/10.3390/toxics13080648/s1, Table S1. pH, Electrical conductivity (EC-µS/cm) and Dissolved Oxygen (DO-mg/L) for *Daphnia magna*, acute immobilization assay at the start and conclusion of the assays after exposure to olive mill wastewater (OMMW) and olive mill wastewater treated with the chemical precipitation technique (CPT-OMWW). Table S2. pH, Electrical conductivity (EC-µS/cm), and Dissolved Oxygen (DO-mg/L) for *Danio rerio*: Fish Embryo Toxicity test (FET) at the start and conclusion of the assays after exposure to olive mill wastewater (OMMW) and olive mill wastewater treated with the chemical precipitation technique (CPT-OMWW). Table S3. pH and Electrical conductivity (EC-µS/cm) in *Folsomia candida* reproduction test after exposure to different concentrations of sludge obtained in the treatment of olive oil industry wastewater by using the chemical precipitation technique. Table S4. pH and Electrical conductivity (EC-µS/cm) in *Enchytraeus crypticus* reproduction test after exposure to different concentrations of sludge obtained in the treatment of olive oil industry wastewater by using the chemical precipitation technique. Table S5. pH and Electrical conductivity (EC-µS/cm) in *Brassica oleracea* after exposure to different concentrations of sludge obtained in the treatment of olive oil industry wastewater by using the chemical precipitation technique. Table S6. pH and Electrical conductivity (EC-µS/cm) in *Lolium perenne* after exposure to different concentrations of sludge obtained in the treatment of olive oil industry wastewater by using the chemical precipitation technique. Figure S1. Germination Index (GI) (%) of (A) *Brassica oleracea* and (B) *Lolium perenne* after exposure to different application rates using different concentrations of sludge in soil (%) obtained in the treatment of olive oil industry wastewater using the Chemical Precipitation Technique. All values are presented as mean ± SE.
