# Supplementary material for: A Comprehensive Ecotoxicological Evaluation of a Treated Olive Mill Wastewater and Obtained Sludge
Source: Toxics. 2025 Jul 30;13(8):648. doi: 10.3390/toxics13080648 (PMC12390398; doi:10.3390/toxics13080648)
Supplement: Supplementary file 1 [file toxics-13-00648-s001.zip › toxics-3790110-supplementary.pdf]

Table S1. pH, Electrical conductivity (EC - µS/cm) and Dissolved Oxygen (DO - mg/L) for *Daphnia magna*, acute immobilization assay at the start and conclusion of the assays after exposure to olive mill wastewater (OMMW) and olive mill wastewater treated with the chemical precipitation technique (CPT-OMWW).

OMWW						
Concentration (%)	Initial			End		
	pH	EC ($\mu S/cm$)	DO (mg/L)	pH	EC ($\mu S/cm$)	DO (mg/L)
0	8.05	558.33	8.61	8.08	562.67	8.41
6.75	7.58	567.33	4.07	7.94	573.33	6.28
12.5	7.42	577	1.21	7.92	584.33	6.15
25	7.23	596.33	0.38	7.86	610	5.23
50	7.02	638.66	0.35	7.77	669.33	2.94
75	6.88	682.66	0.25	7.75	724.67	1.65
100	6.87	729.33	0.34	7.78	783.67	0.99
CPT-OMWW						
	pH	EC ($\mu S/cm$)	DO (mg/L)	pH	EC ($\mu S/cm$)	DO (mg/L)
0	7.57	584	8.01	8.03	793	9.2
6.75	7.7	736	7.93	8.2	856	9.1
12.5	7.67	896	7.94	8.25	1038	9
25	7.61	1210	7.8	8.23	1407	9.2
50	7.55	1879	7.28	8.18	2170	8.6
75	7.54	2660	6.36	8.12	3030	7.9
100	7.68	3290	5.78	8.21	4120	8.7

Table S2. pH, Electrical conductivity (EC - µS/cm) and Dissolved Oxygen (DO - mg/L) for *Danio rerio*: Fish Embryo Toxicity test (FET) at the start and conclusion of the assays after exposure to olive mill wastewater (OMMW) and olive mill wastewater treated with the chemical precipitation technique (CPT-OMWW).

OMWW						
Concentration (%)	Initial			End		
	pH	EC (*µS/cm*)	DO (*mg/L*)	pH	EC (*µS/cm*)	DO (*mg/L*)
0	7.77	830.0	8.21	7.91	810.00	8.01
6.75	7.92	860.0	7.65	7.81	825.00	7.31
12.5	7.91	867.0	6.91	7.77	870.00	5.72
25	7.66	876.0	6.64	7.60	892.00	4.12
50	7.41	792.0	4.92	7.51	823.00	3.65
75	7.06	790.0	2.12	7.51	798.00	1.19
100	6.91	730.0	0.31	7.49	724.00	0.56
CPT-OMWW						
	pH	EC (*µS/cm*)	DO (*mg/L*)	pH	EC (*µS/cm*)	DO (*mg/L*)
0	8.29	800.00	7.91	7.54	812.20	9.50
6.75	8.31	812.00	7.82	7.55	813.40	8.70
12.5	8.33	901.20	7.82	7.58	1023.20	8.80
25	8.38	1241.40	7.72	7.56	1521.34	7.50
50	8.46	1763.45	7.73	7.54	2210.30	8.30
75	8.41	2722.30	7.30	7.53	2982.12	8.30
100	8.35	3331.90	7.61	7.50	4823.30	6.30

Table S3. pH and Electrical conductivity (EC - µS/cm) in *Folsomia candida* reproduction test after exposure to different concentrations of sludge obtained in the treatment of olive oil industry wastewater by using the chemical precipitation technique.

	Initial		End	
Sludge Concentration (%)	pH	EC ($\mu S/cm$)	pH	EC ($\mu S/cm$)
0	5.76	188.73	5.56	190.6
0.5	6.55	142.47	6.64	117.20
1	6.81	129.57	6.28	110.43
2	7.17	173.90	7.66	113.33
4	7.46	187.80	7.72	167.20
8	7.56	244.33	7.60	278.67

Table S4. pH and Electrical conductivity (EC - µS/cm) in *Enchytraeus crypticus* reproduction test after exposure to different concentrations of sludge obtained in the treatment of olive oil industry wastewater by using the chemical precipitation technique.

	Initial		End	
Sludge Concentration (%)	pH	EC ($\mu S/cm$)	pH	EC ($\mu S/cm$)
0	5.5	182.1	5.7	179.3
0.5	6.4	102.6	6.5	101.7
1	6.8	128.9	7.1	106.6
2	7.1	154.9	7.4	122.1
4	7.3	183.9	7.7	157.9
8	7.6	267.7	7.9	223.0

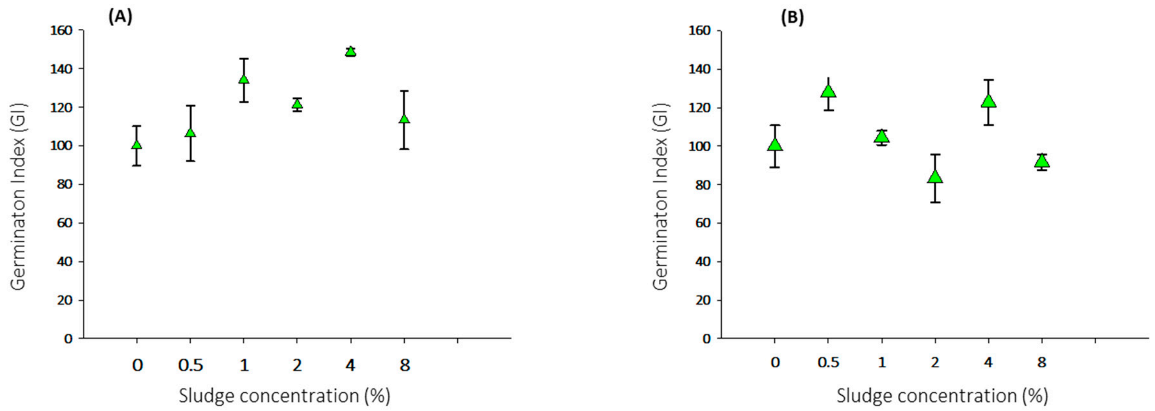

Figure S1. Germination Index (GI) (%) of (A) *Brassica oleracea* and (B) *Lolium perenne* after exposure to different application rates using different concentrations of sludge in soil (%) obtained in the treatment of olive oil industry wastewater using the Chemical Precipitation Technique. All values are presented as mean ± SE.

Table S5. pH and Electrical conductivity (EC - µS/cm) in *Brassica oleracea* after exposure to different concentrations of sludge obtained in the treatment of olive oil industry wastewater by using the chemical precipitation technique.

	Initial		End	
Sludge Concentration (%)	pH	EC (*µS/cm*)	pH	EC (*µS/cm*)
0	5.7	185.9	5.8	241.9
0.5	6.5	120.8	6.6	75.0
1	6.8	128.9	7.1	94.4
2	7.1	164.8	7.3	116.1
4	7.4	186.6	7.6	173.1
8	7.5	255.0	7.8	188.6

Table S6. pH and Electrical conductivity (EC - µS/cm) in *Lolium perenne* after exposure to different concentrations of sludge obtained in the treatment of olive oil industry wastewater by using the chemical precipitation technique.

	Initial		End	
Sludge Concentration (%)	pH	EC (*µS/cm*)	pH	EC (*µS/cm*)
0	6.4	191.7	6.5	230.4
0.5	7.5	98.2	7.6	80.6
1	7.8	124.0	8.2	170.5
2	7.9	140.0	8.4	122.3
4	7.9	180.2	8.4	175.2
8	8.1	226.3	8.4	192.6
